# Cross-validation of the ego dissolution scale: implications for studying psychedelics

**DOI:** 10.3389/fnins.2023.1267611

**Published:** 2023-12-05

**Authors:** Steven Jay Lynn, Charlie W. McDonald, Fiona G. Sleight, Richard E. Mattson

**Affiliations:** Psychology Department, Binghamton University, State University of New York, Binghamton, NY, United States

**Keywords:** ego dissolution, ego dissolution scale, psychedelics, dissociation, depersonalization

## Abstract

**Introduction:**

Ego dissolution, variously called Ego-Loss, self-loss, and ego disintegration, is a hallmark of psychedelic drug use. We cross-validated the 10-item Ego Dissolution Scale, which we developed to assess ego dissolution in everyday life, and we included comparator variables that expanded our original assessment of construct validity.

**Methods:**

Undergraduate college student volunteers (*N* = 527) completed the measures online.

**Results:**

We replicated the original two factor structure (i.e., subfactors: Ego-Loss and Unity/connectedness with others, the world, universe), and we determined that the total score (Cronbach’s α = 0.79) and subfactors (Ego-Loss = 78; Unity = 0.83) possessed adequate-to-good reliability and strong convergent validity (e.g., mindfulness, hallucination-predisposition, sleep variables, personality variables, positive/negative affect transliminality, dissociation/depersonalization), while neuroticism, social desirability did not correlate highly with ego dissolution. We identified distinct patterns of relations of measures associated with the Ego-Loss vs. Unity subfactors.

**Discussion:**

We discuss the implications of the use of the EDS for studying everyday aspects of ego dissolution, the long-term effects of psychedelic use, and the value of using the scale in conjunction with measures of the acute effects of psychedelics.

## 1 Introduction

Ego dissolution is a broad and semantically rich construct variously described as self-loss, Ego-Loss, ego death, and ego disintegration, among other terms (Grof, 1980; Muthukumaraswamy et al., 2013; Carhart-Harris et al., 2014). These sorts of descriptors are admittedly somewhat vague and abstract, as philosophers, experimental psychopathologists, and cognitive scientists have defined the terms “ego” and “self” in diverse ways, the terms lack consensus operational definitions, and “validation and discussion of the ego across disciplines remains ambiguous” (Buchborn et al., 2023, Introduction, para. 2, line 12–13). Still, ego dissolution is a measurable construct (Nour et al., 2016; Stoliker et al., 2022), and the descriptors of ego dissolution arguably have in common reference to one or more of the following experiences of a lack of: (a) a first-person sense of self marked by self-reflective thought; (b) the experience of an ‘I’ as being distinct from others, the world, or the universe; (c) the sense of a corporeal self; and/or (d) awareness of or attunement to a personal narrative or biography (Van Elk and Yaden, 2022; Ego dissolution and affect, paragraph 2; see also Letheby and Gerrans, 2017, narrative self as autobiographical self and Millière et al., 2018). Nour et al. (2016) succinctly defined ego dissolution as a loss of self-referential awareness.

Ego dissolution is a hallmark of psychedelic experiences, which will be the topic of much of our discussion, as psychedelics are both the focus of the collection of papers in which this article appears and well-illustrate how our findings can be applied. Indeed, Millière (2017, p. 4), observes that psychedelic substances provide a platform for appreciating the positive transformational and self-enhancing qualities of ego dissolution as well as more negative aspects of ego dissolution, which can be terrifying as well as blissful. Similarly, Taves (2020) has argued that when the “boundaries of the self are blurred or dissolved,” it can “take on a positive, neutral, or negative valence” (p. 678), which underlines the multifarious nature of ego dissolution experiences (see Sleight et al., 2023).

Yaden et al. (2017) conceptualized ego dissolution via two contrasting facets. The authors term the first facet annihilational, which is characterized by decreased self-salience and diminishment or absence of the sense of self, self-boundaries, and the sense of self in relation to the body. Ego dissolution can be reflected in both (a) negative experiences of “anxious ego-dissolution” (Dittrich, 1998) and in psychotic and delusional episodes, schizophrenia, and depersonalization (i.e., a sense of loss of personhood, Polito et al., 2010; Studerus et al., 2010; Schmid et al., 2015; Simeon et al., 2023); and (b) in positive experiences such as diminished excessive self-focus in anxiety and depression (see Yaden et al., 2017).

The second component, termed relational, is more closely tied to mystical-type experiences of love for others, connectedness with the surroundings, and unity with the universe itself. Not surprisingly, researchers have linked such expansive experiences with positive enduring therapeutic effects, although mystical experiences can evoke a complex mix of positive and negative emotions (Taves, 2020; Yaden and Griffiths, 2021). Additionally, acute negative psychedelic experiences may, in the longer-term, yield to adaptive processing of adverse experiences and ultimately positive outcomes (Carbonaro et al., 2016).

### 1.1 Ego dissolution in everyday life: the ego-dissolution scale

Ego dissolution is best construed as a family or constellation of states (Millière et al., 2018). Because ego dissolution is a multidimensional construct, which can be experienced and studied in diverse ways, it is imperative to facilitate understanding and evaluation of ego dissolution with valid and reliable measures. Further, it is important to determine whether ego dissolution reported on an everyday basis is associated with or is a determinant of the acute and chronic sequela of psychedelic drug use, which have received significant attention in recent years. In a previous study, we developed the 10-item Ego Dissolution Scale (EDS; Sleight et al., 2023; see Supplementary Figure S1) with the aims of (a) assessing potentially positive and negative aspects of ego dissolution in everyday life in a single measure and (b) developing such a measure to complement extant state or acute measures of ego dissolution in relation to psychedelic substances and other antecedents of ego dissolution (e.g., meditation). Measures of the propensity to experience ego dissolution in daily living could (a) assist in pinpointing mechanisms or antecedents of responses to psychedelic drugs by evaluating ego dissolution apart from the acute use of psychedelics and (b) in clinical applications, predict whether psychedelic agents engender or exacerbate psychopathology (i.e., adverse events) following psychedelic use or, conversely, facilitate highly positive and enduring experiences before, during, and after treatment and in other contexts.

Such a brief scale, with a replicable factor structure, would have discernible advantages over previous scales, which have centered exclusively on ego-dissolution in reference to specific antecedent events and have not distinguished positive and negative experiences of ego dissolution separately via the same instrument (e.g., MacLean et al., 2012; Barrett et al., 2015, 2016; Nour et al., 2016). We further suggest that “baseline” everyday experiences of ego dissolution are a potential source of variability in responding to psychedelic substances to be considered in addition to dose of the drug, contextual conditions, expectancies/set, past drug use, and pre-existing psychopathology (Bremler et al., 2023).

To develop the EDS, we started with a large (68-item) pool that included items that spanned disparate aspects of ego dissolution reported in the literature in relation to: (a) the sense of dissolution of the “I” or self (i.e., My “self” disappears and no “me” or “I” is present any longer”); (b) alterations in the experience of the body/embodiment (i.e., “I experience being out of my body”); and (c) unitive experiences (i.e., “I feel at one with the universe”). As the term “ego” may be less understandable, familiar, or “relatable” to participants than the term “self,” we worded items in reference to the “self or ego,” in accord with other scales that index ego dissolution that similarly do not define these constructs. We ascertained that the EDS was reliable and composed of two factors that mapped reasonably well onto Yaden et al.’s (2017) two-component conceptualization.

We (Sleight et al., 2023) determined that self-loss in everyday life could be distilled into two meaningful factors. We labeled the first factor Ego-Loss, which, as the name suggests, was associated with the subjective sense of self-loss and tapped what Yaden et al. (2017) dubbed an annihilation aspect of ego dissolution. This subfactor was associated with negative affect and aspects of dissociative psychopathology (e.g., depersonalization/feelings of loss of personhood, detachment from experiences; dissociative taxon, which reflects severe dissociative psychopathology, neuroticism). In contrast, Unity, a second subfactor, was associated with positive experiences of oneness with others and the universe (i.e., relational component) that are typically associated with mystical-type experiences concomitant with diminished self-salience and enhanced feelings of connectedness (Yaden et al., 2017; Chirico et al., 2022).

As we expected, the scale possessed strong construct validity, particularly in reference to dissociation, which can be defined as the “disruption of and/or discontinuity in the normal integration of consciousness, memory, identity, emotion, perception, body representation, motor control, and behavior” (DSM-5 TR, American Psychiatric Association, 2022, p. 291). We obtained predicted correlations of Ego-Loss with measures of state depersonalization/derealization (depersonalization; derealization: feelings of unreality and/or detachment with respect to surroundings), trait dissociation [i.e., propensity to dissociative experiences of absorption (i.e., tendency to become engrossed in sensory and imaginative experiences), depersonalization/derealization, and forgetting/amnesia in daily life], the dissociative taxon, and state dissociation (i.e., present moment dissociative experiences), which were weakly significant or not significantly related to the Unity subfactor.

These correlations were not surprising, as dissociative experiences generally, and depersonalization/derealization, in particular, are not uncommonly experienced in everyday life (see Lynn et al., 2019). For example, transient symptoms of depersonalization occur with lifetime rates of 26%–74% in the general population (Hunter et al., 2004), and more chronic and disturbing episodes mark depersonalization/derealization disorder in as many as 2% of the general population and 11% among undergraduate students (Yang et al., 2023).

We also, and not surprisingly, secured support for predicted correlations of ego dissolution with (a) mystical-type experiences, particularly with the Unity subfactor; (b) transliminality (i.e., tendency for unconscious material and external stimuli to pass across the threshold of consciousness; Thalbourne, 2001; Thalbourne and Houran, 2005); and (c) positive affect with Unity. We found only small correlations with neuroticism and social desirability. We noted that the EDS could complement the assessment of more acute ego dissolution in response to psychedelics and other antecedent events.

In the current study we provide confirmatory evidence regarding the factor structure of the EDS. We contend that this instrument can be used to advantage in research on psychedelic substances and thus extend the research that we review below.

### 1.2 Psychedelic-induced ego dissolution

We now turn to the relation between ego dissolution and psychedelic drug use, which is the focus of this collection of articles. Tagliazucchi et al. (2016) linked ego dissolution to global brain functional connectivity following ingesting “classical psychedelics,” such as LSD, and Carhart-Harris et al. (2016) linked ego dissolution with decreased activation of the default mode network (DMN) associated with self-representations and the continuity of everyday experiences (Letheby and Gerrans, 2017; Van Elk and Yaden, 2022). Timmermann et al. (2018) reported that consuming dimethyltryptamine (DMT) was also related to ego dissolution, as well as near-death and mystical experiences (see also Liechti, 2017 for research on LSD). Mason et al. (2020) determined that psilocybin increased subjective scores on the Ego Dissolution Inventory (EDI; Nour et al., 2016), and functional changes in brain regions were associated with ego dissolution. These findings combined imply that various traditional psychedelic substances can provide inroads to understanding ego dissolution and similar states.

Non-classic psychedelics can also induce ego dissolution. Li et al. (2023) reported that ketamine infusions, for instance, increase participants’ ratings of ego dissolution on the 5-Dimensions of Altered States of Consciousness (5D-ASC; Dittrich, 1998) scale, and Li et al. (2023) determined that higher scores correlated with activity in multiple subregions in the DMN. Additionally, Kohek et al. (2020) analyzed reports of users of ibogaine—a naturally-occurring plant-based psychedelic–and concluded that ego dissolution is commonly reported after ingestion.

The Ego Dissolution Inventory (EDI; Nour et al., 2016), referred to above, was constructed with specific reference to antecedent psychedelic experiences with the aim to provide a short and reliable measure of ego dissolution potentially relevant to psychedelic-assisted psychotherapy and the understanding of psychosis. The EDI correlated highly with mystical-type experiences (Nour et al., 2016), a construct related to ego dissolution (MEQ; Barrett et al., 2015). Moreover, Martial et al. (2021) reported that EDI scores were associated with reports of self-loss during near-death experiences, and Agin-Liebes et al. (2021) secured online reports of positive effects of ingesting mescaline that were associated with EDI scores. Finally, the EDI documented relations between ego dissolution and reported use of psilocybin, but not other drugs (e.g., cocaine), and vice versa for the ego inflation factor.

Four studies measured ego dissolution with the Ego Dissolution Inventory (EDI; Nour et al., 2016) following ingesting psilocybin at varying doses (Madsen et al., 2020; van Oorsouw et al., 2021, 2022), and all found increases in ego dissolution. Researchers also determined that ayahuasca-induced ego dissolution was related to increased levels of satisfaction with life, changes in affect, and mindfulness (Uthaug et al., 2018). Uthaug et al. (2019) reported that participants with higher ego dissolution scores following a single inhalation of DMT scored lower on measures of depression and stress. Three other studies that used DMT found elevated rates of ego dissolution post-session (Uthaug et al., 2020a,b; Savoldi et al., 2023). However, Uthaug et al. (2021) found no differences between the experimental and placebo group (i.e., inert pill or brew) on total EDI scores. However, as the researchers acknowledge, their conclusions were based on a naturalistic study, and thus may be attributable to lower dose levels than in previous randomized controlled trials. Another EDI study determined that ego dissolution increased after ingesting LSD compared with a comparison condition (i.e., inactive alcohol solution; Wießner et al., 2023).

The studies we reviewed index ego dissolution based on an antecedent event and secure reports regarding an acute event rather than indexing ego dissolution in everyday life. Researchers have devoted insufficient attention to the questions of (a) whether consuming psychedelic substances increases levels of ego dissolution on a long-term or dispositional basis and (b) whether propensities to experience ego dissolution in daily life mediate or moderate responses to psychedelic agents. Indeed, as psychotherapies that integrate psychedelic substances burgeon, the need to examine the diverse sequelae of ego dissolution and its psychological, behavioral, and neurocognitive correlates on an acute and chronic basis in daily life becomes increasingly important. In 2018, Millière et al. contended that studies were not available, at the time, to confirm the enduring effects of psychedelics on ego dissolution. However, they suggested that ego dissolution can be considered trait-like insofar as considerable evidence has documented the relation between alterations in self-consciousness (ego dissolution) and functional and structural changes in brain regions associated with ego dissolution. The fact that ego dissolution is linked with structural changes in brain regions is not dispositive evidence that it is trait-like. Indeed, ego-dissolution experiences might be related to a variety of variables such as psychological stress, anxiety, proneness to psychosis, co-morbid psychopathology, and sleep-related experiences, which we evaluated in our research.

However, research suggests that measures of ego dissolution and psychopathology do remain correlated on at least a limited long-term basis. Van Oorsouw et al. (2022), for example, reported that the EDI and the Beck Depression Inventory (BDI; Beck et al., 2011) correlated both a day and a month after an ayahuasca ceremony, although, at one-year follow-up, the correlations were no longer evident. Similarly, Uthaug et al. (2021) found negative correlations, as expected, between ego dissolution and depression and stress one day after participants completed an ayahuasca ceremony. Nevertheless, these correlations did not persist at the four-week time point. Unfortunately, neither study compared EDI scores at different time points to ascertain their stability nor assessed any demand characteristics of testing. Roseman et al., (2018) determined that decreased symptoms of treatment resistant depression persisted five weeks after a psilocybin therapy session and were mediated by acute experiences of mystical-type experiences and the negative aversive experience of “dread of ego dissolution” reported close to the end of the session and as indexed by the Altered State of Consciousness Questionnaire (Dittrich, 1998; see also Studerus et al., 2010). These findings are consistent with other research (summarized by Roseman et al., 2018) indicating that mystical-type experiences predict positive therapy outcomes. Stoliker et al. (2022) concurred, noting that positive and lasting therapy outcomes are linked with ego dissolution, particularly mystical-type or unity experiences. Clearly, studying the nature and perdurability of the acute effects of ingesting psychedelics with reliable and valid assessment tools remains a research priority.

### 1.3 The current study

The current research was motivated by the assumption that assessing ego dissolution in everyday life can provide a broader and more nuanced picture of the genesis and nature of ego dissolution experiences than reports based on antecedent events alone. In the current study we provide confirmatory evidence regarding the factor structure of the EDS (Sleight et al., 2023), adduce support for the construct validity of the scale, and contend that the EDS can be used to advantage in research on psychedelic substances and thus extend the research we reviewed.

Moreover, we replicate key findings from our first study (Sleight et al., 2023) and also expand the nomological network of variables associated with ego dissolution. We go beyond our initial study with the aim of determining whether measures of personality (i.e., the Big Five personality traits), mindfulness, hallucination predisposition, transliminality, and an array of sleep measures are associated with ego dissolution. We selected these variables and advance predictions below based on our past findings (Sleight et al., 2023), on empirical and/or theoretical links to how psychedelics are experienced or modulated, and on a purely exploratory basis to fill-in gaps in our knowledge base.

One such gap is in the area of ego dissolution and personality. We advance no predictions regarding the link between measures of personality and ego dissolution, apart from openness to experience (referred to as Intellect/Imagination by the measure we utilized in the present study; see Section 2.2.7), which we predict will be associated with ego dissolution. More specifically, psychedelics can alter trait openness and these changes persist over time (MacLean et al., 2011; Lebedev et al., 2016; Wagner et al., 2017). MacLean et al. (2011, p. 1458), for example, found that participants in a high dose psilocybin session who reported a “complete mystical experience” increased in openness from baseline scores at one-year follow-up. In an open-label psychedelic-assisted psychotherapy session of individuals with depression, participants evinced elevated rates of openness from baseline to three-month follow-up (Erritzoe et al., 2018). Further, individuals who report a higher lifetime history of drug use score higher on a measure of transliminality. Thalbourne (2001) suggests that an openness to new experiences may be foundational to both high rates of drug use and transliminality. Finally, Stoliker et al. (2022) contended that “lasting personality change is demonstrated as increased openness to experience,” a “feature of psychedelic effects…also harmonious with observations of reduced connectivity and desegregation of associative resting-state networks” (p. 909).

We included a measure of mindfulness based on our prediction that this construct will be associated with ego dissolution, as awareness of the contents of consciousness facilitates a coherent sense of self. Additionally, meditation typically incorporates mindfulness, and mindfulness is associated with ego dissolution in advanced meditators (Vago and Zeidan, 2016; Schoenberg and Vago, 2019). Moreover, Bagamasbad and Levin (2023) reported a correlation, among comic book convention attendees, of −0.55 between a measure of mindfulness and dissociation, which correlates with ego dissolution (Sleight et al., 2023).

We advanced predictions regarding a number of other variables. We included a measure of hallucination predisposition because it is of potentially significant interest in research on psychedelic drug use. We anticipated that this measure would be related to ego dissolution, as previous studies have reported such a link with dissociation and with problems in reality discrimination, potentially related to a coherent and adaptive sense of self (Varese et al., 2011).

An increasingly impressive corpus of research (see van der Kloet et al., 2012; Lynn et al., 2019) is grounded in the hypothesis that sleep disruptions increase the propensity for dream-like mentation to infiltrate daytime consciousness and thereby engender dissociative experiences (e.g., depersonalization/derealization). Studies have, for example, ascertained a link between dissociation and unusual sleep experiences (e.g., narcolepsy, sleep paralysis). In Sleight et al. (2023), we utilized a single measure of unusual sleep experiences and found the measure to be associated reliably with both dissociation and ego dissolution.

In the current study, we included an omnibus measure (Spoormaker et al., 2005; see Section 2.2.12) that (a) captures both disruptions in the sleep–wake cycle and sleep problems (e.g., insomnia, nightmares, restless leg syndrome) and unusual sleep-related experiences (e.g., narcolepsy) and (b) permits a granular yet exploratory analysis of the link between sleep phenomena and ego dissolution. More specifically, we evaluate a measure of pre-sleep arousal (PSAS-13; Jansson-Fröjmark and Norell-Clarke, 2012) and a well-established measure of diverse sleep experiences and phenomena (i.e., SLEEP-50; Spoormaker et al., 2005). Previous researchers found this measure to be related to dissociative experiences (van Heugten-van der Kloet et al., 2015; Buchnik-Daniely et al., 2021).

As in Sleight et al. (2023), we expect that ego dissolution will be uniquely predicted by transliminality (see also Lange et al., 2019) and sought to replicate this finding. Moreover, we further hypothesize that selected measures, particularly dissociation, will evidence different patterns of correlations with the Ego-Loss versus the Unity factor. First, we predict that measures of dissociation will be more highly correlated with the Ego-Loss factor than with the Unity factor. We are particularly interested in depersonalization/derealization, as we have argued that ego dissolution is a variant of this phenomenon (Sleight et al., 2023). Similarly, Simeon et al. (2023) described depersonalization in terms much akin to ego dissolution as a loss of self or a detachment from the self or aspects of the self. Second, we predict that positive affect will be more highly correlated with the Unity factor than the Ego-Loss factor. Third, we hypothesize that negative affect will be more highly correlated with the Ego-Loss factor than the Unity factor. However, we advance no specific predictions regarding the link between hallucination predisposition, transliminality, and mindfulness (i.e., paying purposeful nonjudgmental attention to the contents of consciousness, see Section 2.2.4) with respect to differential responses to the factors of ego dissolution. We will also determine which variables account for unique variability in ego dissolution beyond measures of dissociation, including neuroticism and social desirability.

## 2 Method

### 2.1 Participants

We recruited 527 undergraduate students (66.8% female, 33.2% male; *M_age_* = 19.20, *SD* = 1.08) from an online participant pool in exchange for course credit. Most of the sample identified as Caucasian (66.8%), with 18.4% identifying as Asian, 7.6%, as multiple identities or reported their identity was not listed, 6.5% as Black, 0.4% identified as Native American, and 0.4% as Native Hawaiian or Pacific Islander. The University IRB approved this study.

### 2.2 Measures

#### 2.2.1 Clinician-administered dissociative states scale (CADSS)

The CADSS contains 19 self-report items (used in the present study) and eight observer-scored items assessing state dissociation at the time of administration (Bremner et al., 1998). Participants rated the extent to which they were experiencing each item currently, from 0 (*Not at all*) to 4 (*Extreme*). The CADSS is sensitive to experimental manipulations of dissociative states (e.g., van Heugten-van der Kloet et al., 2018) and exhibited good internal consistency in our study (Cronbach’s α = 0.89).

#### 2.2.2 Dissociative experiences scale-II (DES-II)

The 28-item DES-II self-report questionnaire assesses the frequency of dissociative experiences in daily life across absorption, depersonalization/derealization, and amnestic dissociation subscales (Carlson and Putnam, 1993). We did not analyze the absorption and amnesia subscales in our study because they did not contribute unique variance to the prediction of ego dissolution variables in our previous study (Sleight et al., 2023). Participants indicate the percentage of time each item occurs in daily life from 0% (*never*) to 100% (*always*). The DES-II has good convergent validity, with higher scores associated with more severe dissociative disorders (Lyssenko et al., 2018). In our study, the DES-II exhibited excellent internal consistency (Cronbach’s α = 0.94), and its subscales possessed good internal consistency (Cronbach’s αs from 0.82 to 0.86).

The DES-T is comprised of eight DES-II items that were identified through taxometric analyses and was calculated to assess pathological dissociation (Waller et al., 1996). The items thus overlapped with the DES-II. The DES-T also possessed good internal consistency in our study (Cronbach’s α = 0.84).

#### 2.2.3 Ego dissolution scale (EDS)

The 10-item EDS is a self-report questionnaire quantifying trait-like propensities towards self-loss experiences (Sleight et al., 2023). Participants rate the percentage of time each item occurs in their daily life from 0% (*never*) to 100% (*always*). Two subscale scores were derived from the termed ‘Ego-Loss,’ mapping largely on to what Yaden et al. (2017) described as the annihilational (i.e., subjective sense of loss of self or “I”) component of ego dissolution and another assessing the relational component (i.e., Unity). The EDS demonstrated acceptable internal consistency (Cronbach’s α = 0.79), and the Ego-Loss and Unity subscales demonstrated good and acceptable internal consistency, respectively (Cronbach’s αs of 0.83 and 0.78).

#### 2.2.4 Five facet mindfulness questionnaire (FFMQ)

The 39-item FFMQ self-report questionnaire quantifies mindfulness in daily life across five facets: observing, describing, acting with awareness, nonjudging (e.g., being aware of but not judging the contents of consciousness), and non-reactivity (Baer et al., 2006). Participants rate the extent to which each item is true for them, from 1 (*Never or very rarely true*) to 5 (*Very often or always true*). High FFMQ scores are associated with meditation experience and psychological well-being (Baer et al., 2008). In Sleight et al. (2023), the full scale demonstrated good internal consistency (Cronbach’s α = 0.83), and its subscales exhibited acceptable (Non-reactivity; Cronbach’s α = 0.76) to excellent (Nonjudging; Cronbach’s α = 0.91) internal consistency.

#### 2.2.5 Launay-Slade hallucination scale-revised (LSHS-R)

The 12-item LSHS self-report scale assesses a predisposition to hallucinations in healthy and clinical populations that encompasses vivid daydreams, tendency toward hallucinatory experiences, and subjective externality of thought (Bentall and Slade, 1985; Aleman et al., 2001). Participants rate how applicable each item is to them from 0 (*certainly does not apply to you*) to 4 (*certainly does apply to you*). The LSHS-R demonstrated good internal consistency in our study (Cronbach’s α = 0.86).

#### 2.2.6 Marlowe-Crowne social desirability scale form C (M-C form C)

The 13-item (True/False) M-C Form C self-report questionnaire determines the extent to which a participant represents themselves in a socially desirable manner (Reynolds, 1982). Participants respond to questions that are infrequently endorsed by participants responding honestly. The M-C Form C demonstrated questionable internal consistency in our study (Cronbach’s α = 0.63).

#### 2.2.7 Mini-IPIP

The 20-item Mini-IPIP self-report questionnaire assesses personality based on the Big Five model (Donnellan et al., 2006). The Mini-IPIP was derived from the 50-item International Personality Pool-Five-Factor Model measure (Goldberg, 1999) and contains four items assessing each personality trait. Participants rate how personally applicable each item is from 0 (*Very inaccurate*) to 5 (*Very accurate*). The Mini-IPIP demonstrates acceptable psychometric properties (Donnellan et al., 2006; Cooper et al., 2010; Baldasaro et al., 2013). The Mini-IPIP scales demonstrated questionable (Intellect/Imagination; Cronbach’s α = 0.61) to good (Extraversion; Cronbach’s α = 0.82) internal consistency in our study.

#### 2.2.8 Neuroticism subscale of the NEO five-factor inventory (NEO-FFI)

The 12-item neuroticism subscale of the NEO-FFI assesses trait negative affectivity (Costa and McCrae, 1992). Participants rated items on a Likert scale from 1 (*strongly disagree*) to 5 (*strongly agree*). The NEO-FFI is valid and reliable (e.g., McCrae and Costa, 2004; Anisi, 2012; Perera et al., 2015) and exhibited good internal consistency in our study (Cronbach’s α = 0.82). We selected this measure vs. the index from the Mini-IPIP as our measure of general negative affect because it is more widely reported in the literature.

#### 2.2.9 Positive and negative affect schedule (PANAS)

The 20-item PANAS self-report measure quantifies affect at the time of administration (Watson et al., 1988). Participants rate emotion words (e.g., Ashamed) on a Likert scale from 1 (*Very slightly or not at all*) to 5 (*Extremely*). The PANAS exhibits convergent and discriminant validity with measures of general distress, dysfunction, and psychopathology (Watson et al., 1988; Crawford and Henry, 2004). The positive and negative scales possessed excellent internal consistency in our study (alphas of 0.91 and 0.90, respectively).

#### 2.2.10 Pre-sleep arousal scale (PSAS-13)

The 13-item PSAS-13 self-report questionnaire assesses typical arousal levels while attempting to sleep (Jansson-Fröjmark and Norell-Clarke, 2012). Participants rate 13 items related to cognitive (e.g., “Thoughts keep running through your head”) and somatic (e.g., “Dry feeling in mouth or throat”) arousal from 1 (*Not at all*) to 5 (*Extremely*). The PSAS-13 can discriminate between individuals with poor sleep or insomnia and those with normal sleep (Jansson-Fröjmark and Norell-Clarke, 2012; Vochem et al., 2019). The PSAS-13 demonstrated excellent internal consistency in our study (Cronbach’s α = 0.90).

#### 2.2.11 Revised transliminality scale (RTS)

The RTS self-report measure assesses behaviors, experiences, and attitudes across 17 True/False questions related to transliminality (Lange et al., 2000). Transliminality is theorized to be an underlying factor across various traits such as propensity to mystical experiences and fantasy proneness (Thalbourne et al., 1997; Fleck et al., 2008). The RTS evinces convergent validity with related constructs (e.g., openness to experience; Lange et al., 2019) and exhibited good internal consistency in our study (Cronbach’s α = 0.81).

#### 2.2.12 SLEEP-50

The SLEEP-50 is a comprehensive self-report measure of sleep-related difficulties, including sleep disorders (e.g., sleep apnea) and functional impairment related to poor sleep environment or quality of sleep (Spoormaker et al., 2005). Participants rate the extent to which each statement has applied to them over the past 4 weeks from 1 (*Not at all*) to 4 (*Very much*). Nine subscale scores include Sleep Apnea, Insomnia, Narcolepsy, Restless Legs/Periodic Limb Movements of Sleep, Circadian Rhythm Sleep Disorder, Sleepwalking, Nightmares, Factors Influencing Sleep, and the Impact of Sleep Complaints on Daily Functioning. The SLEEP-50 has good psychometric properties among clinical and nonclinical samples (Spoormaker et al., 2005). In our study, the internal consistency of the subscales ranged from poor (Circadian Rhythm Sleep Disorder; Cronbach’s α = 0.59) to good (Sleep Complaints on Daily Functioning; Cronbach’s α = 0.84).

### 2.3 Procedure

We advertised our study on SONA, a participant management software, and we presented our research titled “Assessing the Self and Experiences” to participants as an examination of mood, personality, beliefs, and personal experiences. After we obtained informed consent, participants completed a demographic survey followed by randomized self-report measures. The survey was designed and implemented through Qualtrics. The median duration of participation was 62 min.

### 2.4 Analyses plan

#### 2.4.1 Missing data

We performed statistical analyses using SPSS version 28. We removed individuals who exceeded 20% missing data (*n* = 32) to yield a final sample of 527 participants. We used expectation maximization to estimate missing values (Schafer and Graham, 2002) and calculated total scores for each measure. Note that the total EDS score will subsequently be referred to as “Ego Dissolution.” We utilized conservative Bonferroni-corrected alpha levels throughout to account for the numerous analyses conducted. All correlational findings described were significant at a Bonferroni-corrected alpha level of 0.00000082.

#### 2.4.2 Confirmatory factor analysis

We first performed a confirmatory factor analysis of the EDS in AMOS Graphics 28. We specified the proposed two-factor model with four and six items loading onto the Unity and Ego-Loss latent factors discretely. The Unity and Ego-Loss factors were allowed to intercorrelate and the model was estimated using Maximum Likelihood Estimation. Model fit to the data was evaluated using the Confirmatory Fit Index (CFI), the Standardized Root Mean Residual (SRMR), and the Root Mean Square Error of Approximation (RMSEA). The cutoff for acceptable fit is greater than 0.90 for the CFI, and less than 0.08 and 0.10 for the SRMR and the RMSEA (see MacCallum et al., 1996; Hu and Bentler, 1999). Standardized factors loadings will be reported. For comparison, we also ran a model positing only a single factor on which all of the EDS items loaded. We then explored a bifactor model positing one overall construct predicting variance across all items, with two subconstruct tied to either the Unity or Ego-Loss items.

#### 2.4.3 Correlation analyses

We conducted correlation analyses using Pearson’s *r* and Cohen’s guidelines (Cohen, 1988) to label effect sizes (0.10 = small; 0.30 = moderate; 0.50 = large). We compared correlation coefficients using the test of the difference between two dependent correlations with one variable in common computer software (Steiger, 1980; Lee and Preacher, 2013).

#### 2.4.4 Regression analyses

We employed a stepwise (i.e., statistical) regression approach to model our set of predictors. Although stepwise regression has some limitations (e.g., capitalization on sampling error; Thompson, 1995), the current application is justified given the exploratory nature of the analyses. More specifically, ego dissolution is not a well-understood construct, and research on its nomological domain is limited. However, there are a wide range of candidate variables that pertain to both ego dissolution and psychedelic experience, with little in the way of theory to guide the selection of some variables over others. As such, an approach that samples a large range of candidate predictors that are then honed mathematically into a smaller, uniquely predictive set is presently justified. The stepwise approach currently used enters in the strongest overall predictor first, followed by predictors of decreasing associative strength. We ran three stepwise multiple regressions evaluating the total scale and each of the two subscales in separate models. Linear transformations were applied to any variables requiring correction for non-normality. Preliminary analyses were run to identify high-influence cases, which were removed model-wise. The Variance Inflation Factor (VIF) was used to identify any problematic multicollinearity. Residual analyses were then performed to identify possible heteroskedasticity. *R^2^* will be reported for the final block of the model, but an estimate adjusted for the initial *df* (i.e., accounting for the size of the initial slate of predictors) will also be provided. We report standardized betas (β) and semi-partial correlations (*sr*) in order to facilitate the comparison of the predictors within each model. Unstandardized regression weights, standard errors, 95%CI, *t* and *p*-values will be presented alongside the bivariate *r* for each predictor in the Supplementary material.

## 3 Results

### 3.1 Confirmatory factor analysis (one-factor and bifactor) and reliabilities

**Figure 1:**
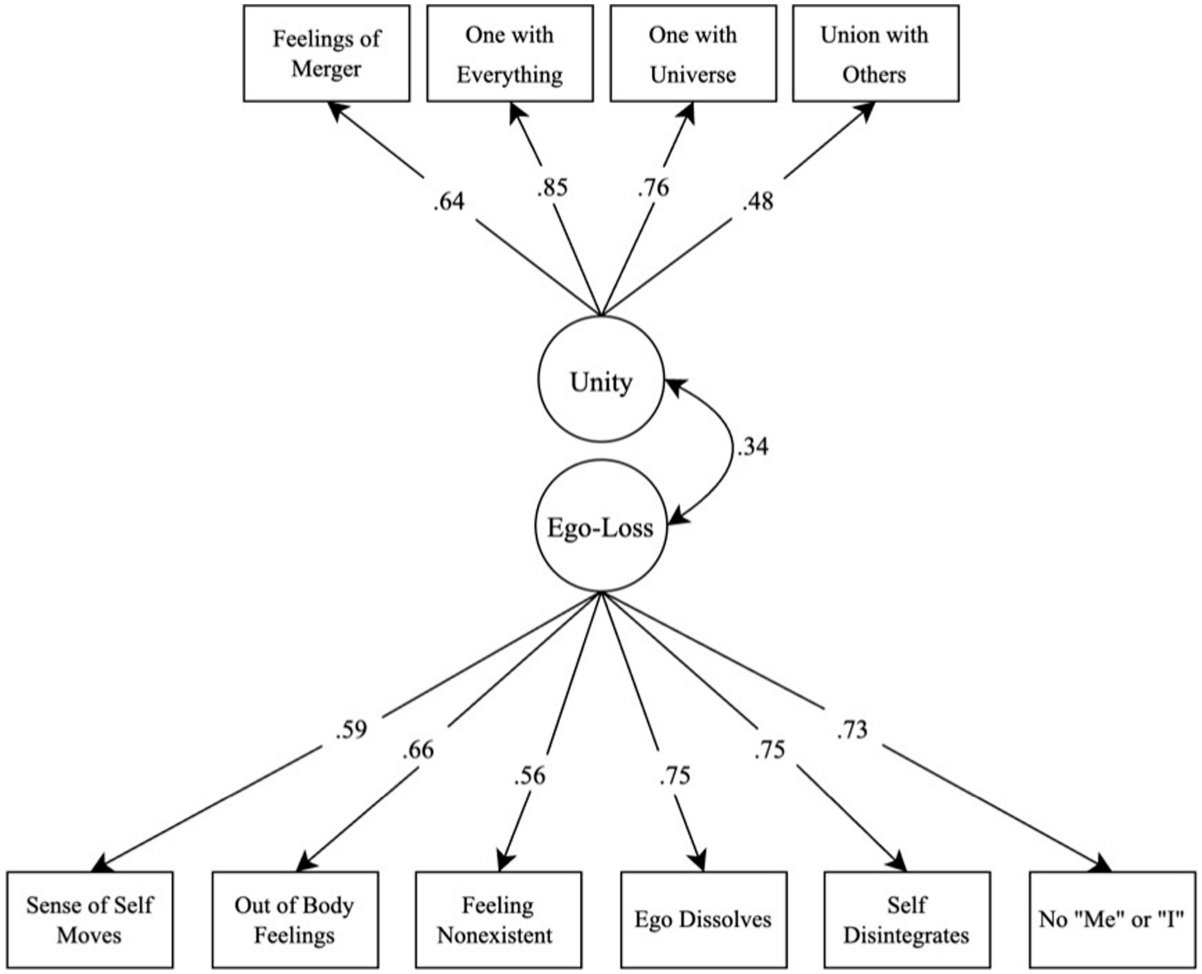
Standardized factor loadings from the factor analysis confirming the two-dimensional structure of the EDS.

We hypothesized that the EDS items would conform to the two-factor structure identified by Sleight et al. (2023), with a significant positive correlation between the Unity and the Ego-Loss factors. As hypothesized, the model provided a good fit to the data, χ^2^(34) = 169.484, *p* < 0.001, CFI = 0.925, SRMR = 0.067, and RMSEA = 0.087. All item loadings were statistically significant, suggesting that each item confers information relevant to the underlying latent variable to which it is theoretically assigned. Estimates of standardized loadings are presented in Figure 1. The Ego-Loss and Unity dimensions were significantly correlated, such that higher elevations on the Unity dimension corresponded to higher elevations on Ego-Loss, and vice versa for lower elevations, which is consistent with previous research. These results overall replicate the exploratory factor analyses conducted by Sleight et al. (2023) and thus provide strong evidence that the EDS covers both hypothesized components of ego dissolution.

The modeling positing only a single factor for the EDS was a poor fit to the data across criteria, *x^2^*(35) = 673.99, *p* < 0.001, CFI = 0.644, SRMR = 0.140, and RMSEA = 0.186. The best fitting model was the bifactor structure, *x^2^*(35) = 55.680, *p* < 0.001, CFI = 0.983, and RMSEA = 0.048 (SRMR cannot be calculated for bifactor models). This model retained the separate Unity and Ego-Loss latent factors; but, in lieu of modeling their intercorrelation, specified an omnibus factor onto which all of the items loaded. This overall suggests that the EDS items together assess the broad latent construct of ego dissolution, as well as provide separate assessments of both Ego-Loss and Unity experiences separately.

Reliabilities of the total scale and subscales ranged from acceptable-to-good. The full scale exhibited acceptable internal consistency (Cronbach’s α = 0.79). The Ego-Loss and Unity subscales exhibited acceptable and good internal consistency, respectively (Cronbach’s αs of 0.78 and 0.83) and were both strongly positively associated with the total scale (*r* = 0.80 and 0.70, respectively) and weakly correlated with each other (*r* = 0.26).

### 3.2 Correlation analyses

Supplementary Table S1 presents our correlational findings. As we predicted, Ego Dissolution was strongly positively correlated with measures of trait dissociation, trait depersonalization/derealization, and pathological dissociation (*r*s = 0.53–0.54). Likewise, moderate correlations of Ego Dissolution were observed with state dissociation, hallucination predisposition, transliminality, and sleepwalking (*r*s = 0.33–0.41). Finally, Ego Dissolution had small yet significant positive associations with positive and negative affect, mindful observing, and a variety of sleep measures, including symptoms of sleep apnea, symptoms of narcolepsy, symptoms of restless leg syndrome/PLMD, and factors that affect sleep quality.

We examined correlations of Ego-Loss and Unity with other variables. As predicted, Ego-Loss was strongly positively correlated with trait dissociation, trait derealization/depersonalization, pathological dissociation, and state dissociation. Ego-Loss was moderately positively correlated with negative affect, hallucination predisposition, transliminality, pre-sleep arousal, sleep apnea symptoms, insomnia symptoms, narcolepsy symptoms, restless leg syndrome/periodic limb movements, sleepwalking, and factors that affect sleep quality; and moderately negatively associated with mindful acting with awareness. Ego-Loss evinced small positive correlations with neuroticism (both Mini IPIP and NEO-FFI), circadian rhythm dysfunction, nightmares, and functional impairment due to poor sleep, as well as weak negative correlations with trait mindfulness and mindful describing and nonjudging. Unity was moderately positively associated with positive affect, as expected, and had small yet significant positive associations with extraversion, trait mindfulness, including the facet observing, and transliminality.

We first compared correlations of variables with respect to Ego-Loss and Unity. All correlations evinced statistically significant differences across the two factors with the exceptions of intellect/imagination, transliminality and mindful observing and non-reactivity, which were comparable in magnitude. However, as predicted, Ego-Loss was significantly more strongly associated with trait dissociation, trait depersonalization/derealization, and pathological dissociation (*z* = 10.88, *p* < 0.001) than was Unity. Similarly, all sleep-related variables were significantly more associated with Ego-Loss (*p*s < 0.001) than with Unity. Finally, positive affect was significantly more strongly associated with Unity (*z* = −4.09, *p* < 0.001), whereas negative affect was significantly more associated with Ego-Loss (*z* = 9.12, *p* < 0.001), as hypothesized. Supplementary Table S2 presents these findings in more detail.

Another aim of our study was to assess the potential convergence of Ego-Loss with trait depersonalization/derealization. In comparing the magnitude of correlations between Ego-Loss and trait depersonalization/derealization with other variables included in our study, no correlations exhibited statistically significant differences. Namely, Ego-Loss and trait depersonalization/derealization demonstrated equivalent statistical associations with all other variables included in our study. Conversely, the relation between Unity and trait depersonalization/derealization and other variables was primarily divergent, with the exception of intellect/imagination, mindful observing and nonreactivity, transliminality, and nightmares. Supplementary Table S3 presents these findings in more detail.

### 3.3 Regression analyses

Our focus was to characterize the EDS in terms of the most powerful set of unique predictors among several theoretically-related correlates. Regarding the latter, we sought to replicate certain prior findings on the EDS. We also once again included the DES-II as well as the subscale of depersonalization and the dissociative taxon scale (DES-T), as well as the CADSS measure of state dissociation. We also included measures of constructs that tap other theoretically-relevant but novel constructs. The standardized regression weights and significance values for each predictor across three models are presented in Supplementary Tables S4–S6, and the with the semipartial correlations are included in the text below.

We first evaluated the total scores on the EDS as the criterion. The strongest predictor of the total score was transliminality, *sr* = 0.18, followed by pathological trait dissociation, *sr* = 0.11. The total score also associated significantly with personality traits, including trait extraversion, *sr* = 0.12, trait conscientiousness, *sr* = 0.10, and trait neuroticism, *sr* = −0.12. Mindful observing, *sr* = 0.12, and trait depersonalization/derealization, *sr* = 0.10, made similar unique contributions. The set of predictors overall accounted for 41.2% of the overall variance, with an adjusted *R^2^* equal to 0.404.

With respect to the subscales, trait derealization/depersonalization, *sr* = 0.19, and state dissociation, *sr* = 0.13, were the two primary associates of Ego-Loss, with pathological dissociation having smaller though significant unique associations, *sr* = 0.07. State negative affect, *sr* = 0.10, and circadian rhythm disruption, *sr* = 0.09, were also uniquely predictive of Ego-Loss, with higher elevations associating with a higher level of Ego-Loss. Finally, mindful describing was negatively associated with Ego-Loss experiences, *sr* = −0.07, with the total set of predictors accounting for 61.1% of the variance in Ego-Loss (*R^2^*_adjusted_ = 0.606).

Regarding the Unity subscale, the strongest predictor was state positive affect, which associated positively, *sr* = 0.17, followed by transliminality, *sr* = 0.14, which was also positively associated. Trait extraversion, *sr* = 0.20, pathological trait dissociation, *sr* = 0.19, mindful observing, *sr* = 0.18, social desirability, *sr* = 0.10, and trait agreeableness, *sr* = 0.07, were all positively and uniquely associated with Unity. Several scales carried negative associations with Unity: namely, trait neuroticism, *sr* = −0.11, pre-sleep arousal, *sr* = −0.09, and state negative affect, *sr* = −0.08. The overall set of predictors accounted for 34.6% of the variation (*R^2^*_adjusted_ = 0.333).

## 4 Discussion

Our study is the first to successfully cross-validate a measure of ego dissolution in everyday life that captures positive and negative subjective experiences related to the broader literature regarding the diverse ways that ego dissolution can be manifested (i.e., Ego-Loss and Unity). Our current study verifies and extends the factor structure of the EDS, replicates important findings, expands our understanding of the domain of the construct of ego dissolution, and confirms the link of ego dissolution with the construct of dissociation, particularly depersonalization/derealization, as we expected.

### 4.1 Psychometric properties

The 10-item EDS was reliable: The total score (Ego Dissolution) and subscales (Ego-Loss and Unity), which we derived from a confirmatory factor analysis, exhibited acceptable-to-good internal consistency, and we replicated the original factor structure (Sleight et al., 2023). Ego Dissolution proper (i.e., total score) is defined by the overlap between Ego-Loss and Unity experiences, but these subdimensions represent a broader construct that does not necessarily fall within their parent domain. More specifically, it may be possible to experience Unity without Ego-Loss (e.g., cognitive appreciation for one’s connection to the broader universe) and Ego-Loss without Unity (e.g., a “bad trip”), but Ego Dissolution proper must reflect the combination of these experiences. Nevertheless, aspects of those experiences do not necessarily intersect. As in our original study, the Ego-Loss and Unity factors were only moderately correlated, signifying that one can experience Unity of Ego-Loss without the other, and justifies the examination of the total score alongside the two separate subscales we discuss below. We were able to predict substantial sources of unique variance with the variables we selected in terms of predicting all three measures of ego dissolution (Ego Dissolution overall variance accounted for = 41.2%; Ego-Loss overall variance accounted for = 61.1%; Unity overall variance accounted for = 34.6%).

Further psychometric work on the EDS is warranted to potentially increase the number of items that correspond to each subfactor to increase the reliability of the instrument, and future researchers could adapt the EDS to assess acute reactions to antecedent events. Moreover, our conclusions are limited to generalizability to a college student population, and additional studies are necessary to explore differences in responses to the measure as a function of diverse demographic and diagnostic groups as well as cultural influences. Given the empirical link between dissociation and ego dissolution, it would be particularly worthwhile to determine whether high dissociation predicts particularly intense or negative responses to psychedelic agents on an acute or chronic basis.

Test–retest data will be essential to obtain across varying time intervals. Research that evaluates acute responses to psychedelics in relation to general measures of the phenomenology of consciousness (e.g., Pekala et al., 1986; Phenomenology of Consciousness Inventory) and studies that compare different measures of ego dissolution to further assess the construct would be worthwhile to assess convergent validity. Additionally, we suggest that researchers explore neurocognitive differences across people who differ in dispositional (e.g., frequency and intensity of episodes of ego dissolution) aspects of ego-dissolution compared with dissociation and across the two subscales to assess discriminant validity. Moreover, researchers and clinicians could administer the EDS before and after psychedelic drug use, or other methods of inducing or assessing ego dissolution, to assess changes following targeted interventions in and apart from psychotherapy.

### 4.2 Predictions and relations among variables

As in our initial study, and as we anticipated, Ego Dissolution and Ego-Loss were associated with all measures of dissociation (i.e., trait dissociation, depersonalization/derealization, pathological dissociation, and state dissociation), albeit substantial variance between measures of ego dissolution and dissociation remains to be accounted for. Notably, trait depersonalization/derealization and pathological dissociation correlated highly (*r* = 0.67 current study; *r* = 0.71, Sleight et al., 2023) with Ego-Loss, and we found equivalent correlations with Ego-Loss and depersonalization/derealization across every comparator variable (see Supplementary Table S3), providing strong evidence of convergence between these measures, suggestive of a shared domain of subjective experience. However, it remains to be determined whether (a) tendencies to dissociate would be predictive of acute ego dissolution, as measured by the EDI (Nour et al., 2016), for example, and (b) whether, similar to dissociative experiences, ego dissolution experiences in everyday life are related to stress-related intraindividual differences as is the case in dissociation (Soffer-Dudek, 2017).

However, ego dissolution cannot be reduced to dissociation: No measure of dissociation exceeded a correlation of *r* = 0.21 with Unity (compared with *r* = 0.15, Sleight et al., 2023). Unity perhaps more represents a tendency to connect or “associate” rather than dissociate. Indeed, the majority of correlations of Unity and other variables differed significantly from correlations with both Ego-Loss and depersonalization/derealization. These findings imply that the association of dissociation with ego dissolution is largely constrained to more pathological aspects of dissociation.

In multiple regression analysis, the two primary associates of Ego-Loss were trait derealization/depersonalization and state dissociation. Pathological dissociation and state negative affect, circadian rhythm disruption, and mindful describing also were unique predictors of Ego-Loss, with these variables altogether accounting for more than 60 percent of the variance in Ego-Loss, as noted above. In summary, our findings buttress our contention that Ego-Loss, can be construed as a variant of depersonalization/derealization but the latter is not highly associated with unitive experiences.

We also found evidence for links between transliminality and Ego Dissolution, Ego-Loss, and Unity, which is not surprising given (a) that mystical-type experiences are highly correlated with transliminality (Evans et al., 2019) and (b) our contention that highly diverse cognitions and emotions pass in out of consciousness in conjunction with attenuated cognitive control associated with Ego-Loss and Unity. In fact, transliminality emerged as the strongest unique predictor of the EDS total score.

We included measures of sleep, hallucination predisposition, personality, and mindfulness, which we did not evaluate previously. In Sleight et al. (2023), we documented an association between a single measure of unusual sleep experiences and Ego Dissolution and Ego-Loss. However, our current study conducted a more comprehensive analysis of ego dissolution and sleep. On average, Ego-Loss was more highly positively correlated with sleep measures, relative to other measures of ego dissolution. The obtained correlation of *r* = 0.47 of Ego-Loss with narcolepsy, a proxy for unusual experiences in our initial study, is generally consistent with the previously observed correlation of *r* = 0.36 with unusual sleep experiences (Sleight et al., 2023).

As in Sleight et al. (2023), correlations of the sleep measures with Unity were not significant and, in some cases, negative and statistically lower than the link with both Ego-Loss and Ego Dissolution. We thus provided confirmatory evidence for the discriminant validity of the Unity measure in terms of a broad range of sleep variables, which begs the question of a link of sleep variables with acute responses to psychedelics.

We also found support for the hypothesized association of ego dissolution and hallucination predisposition. Hallucination predisposition was positively linked with Ego Dissolution and Ego-Loss but was not significantly associated with Unity. Researchers could profitably study hallucination predisposition with regard to the nature and intensity of acute hallucinatory responses to psychedelics.

We secured complex findings regarding mindfulness and ego dissolution; yet, overall, we confirmed a hypothesized relation between these constructs. As expected, Ego-Loss was negatively associated with trait mindfulness, describing, acting with awareness, and nonjudging. In contrast, Unity was positively associated with both trait mindfulness and describing, and not associated with nonjudging, providing evidence of discriminant validity. Mindful acting with awareness was negatively associated with Ego Dissolution and Ego-Loss, but not associated with Unity.

Only mindful observing was positively associated with all three measures of ego dissolution and contributed unique variance to predicting ego dissolution, whereas nonreactivity was not associated with any measure of ego dissolution, proving to be the least discriminant measure of mindfulness. Further research is necessary to determine why mindful observing would be associated with both Unity and Ego-Loss aspects of ego dissolution. Perhaps observing the contents of consciousness increases awareness and sensitivity to the experience of Ego-Loss, whereas nonreactivity appears to be independent of ego dissolution.

Our findings regarding personality variables represent a significant contribution to elaborating the construct validity of ego dissolution. Contrary to our prediction, intellect/imagination was not associated with ego dissolution. Notably, this subscale of the Mini-IPIP was the least internally reliable among the five, implying that future studies should include more extensive and reliable assessments of openness.

The fact that extraversion was the strongest predictor of Unity, and agreeableness contributed unique variance in a multiple regression analysis, suggests that extraverted individuals may be predisposed to interpersonal connection and/or, alternately, that a sense of unity fosters these personality attributes. Extraversion also predicted the total EDS score, probably due to the contribution of Unity items, and conscientiousness predicted a small amount of unique variance in predicting Ego Dissolution. The direction of causality, including the determination of potential recursive relations among variables merits clarification in future research.

Negative associations with trait neuroticism and state negative affect underscore the link of positive emotions with the construct of Unity. We speculate that “unity” implies a positive tendency to *associate* or connect aspects of the self with others, the surroundings, and the universe writ large in a manner that counteracts or nullifies more negative aspects of dissociation. Indeed, positive affect was significantly correlated with Unity but not with Ego-Loss, and negative affect was significantly correlated with Ego-Loss but was not correlated with Unity.

After extraversion, the strongest predictor of Unity was pathological trait dissociation, followed by mindful observing, positive affect, transliminality, social desirability, and trait agreeableness. Scales with negative associations with Unity included negative affect, pre-sleep arousal, and trait neuroticism. Combined, these predictors accounted for almost 35 percent of the variation in Unity, as noted above. The most surprising finding was pathological trait dissociation having a positive association with unity. One explanation pertains to the “perils of partialling;” that is, the notion that measures may no longer represent their original construct after being conditioned on related measures. A more interesting speculation is that Unity experiences encompass merger experiences between the self and others, which sometimes can be a component of psychopathology (e.g., borderline personality disorder). As in Sleight et al., 2023, neither social desirability nor neuroticism were influential determinants of the pattern of findings obtained.

### 4.3 Limitations and future directions

In this section, we address limitations of our work and provide suggestions for future research. One limitation is the lack of data capturing history and current use of psychedelics and data regarding events that precede ego dissolution. Our study would have benefited from an examination of participant retrospective reports of psychedelic experiences and a determination of the extent to which such reports mapped onto our measure.

Another limitation is that we did not incorporate variables to distinguish ego dissolution from allied constructs such as mystical experiences (Stace, 1960; Hood, 1975) and awe, which is a proposed mechanism of action in psychedelic-assisted psychotherapy linked with ego dissolution (Hendricks, 2018; see also Van Elk and Yaden, 2022). Taves (2020) went so far as to suggest that mystical experiences may be a variant of ego dissolution, as in the former experiences, the sense of self can fall away entirely, engendering a sense of unity with one’s surroundings. Accordingly, we recommend that future EDS studies evaluate these constructs across different modalities of inducing ego dissolution (e.g., psychedelic use, meditation).

In our scale, as in other scales, the terms “ego” and “self” may be open to interpretation and contribute to variability in participant responses. Future studies could interview participants (see Lindström et al., 2022) with regard to their conceptualization of these terms, their similarities and differences, and investigate the phenomenology of ego dissolution in relation to different aspects of the self, as described in the literature (e.g., minimal self; that is, pre-reflective experience of being a self, Blanke and Metzinger, 2009; the social self in relation to others; the sense of self in relation to the body; and the narrative/autobiographical/reflective self; see Letheby and Gerrans, 2017; Millière, 2017). Although the EDS does not separately examine these different aspects of the self, empirical scales could be developed that disambiguate these potentially discriminable aspects of ego dissolution in assaying acute effects of psychedelics.

Few studies have explored ego dissolution longitudinally (e.g., van Oorsouw et al., 2022). Due to the cross-sectional nature of our research, we could not probe the continuity of ego dissolution over time and derive causal claims among variables. A critical need exists to examine the stability of everyday ego dissolution and whether more elevated rates of Ego-Loss and Unity, for example, are related to differential outcomes over time. Longitudinal research could explore whether Ego-Loss in everyday life “opens the door” to crystallized experiences of unity and/or negative experiences in psychedelic therapy and whether acute psychedelic experiences (e.g., “good” and “bad” trips) are modulated by variables (e.g., hallucination predisposition, dissociation, sleep experiences, emotion regulation, neurocognitive variables) we determined are empirically linked with ego dissolution. It would also be worthwhile to explore the link between ego dissolution and hallucinogen persisting perception disorder and the potential of the EDS to be used in conjunction with screening for this condition (Martinotti et al., 2018). Studies that disambiguate dissociation and ego dissolution should also be accorded a high priority.

There is ample space for researchers to explore associations and differences between ego dissolution experiences in daily life and acute responses in the depths of a psychedelic experience and following the use of psychedelics. These latter experiences probably differ greatly in their impact, richness, hallucinatory aspects, intensity, perdurability, self-awareness, and insights regarding the self and may differ in their correlates with positive changes in personality, psychopathology, and resilience to stress. Phenomenological research based on open-ended and semi-structured interviews would be a very useful starting point in this line of research and go beyond extant accounts based largely on anecdotal reports. Studies that delineate more precisely what experiences qualify as ego dissolution vs. dissociation would also be welcome.

Ego dissolution ranges from highly positive unity experiences, which might inoculate against psychopathology and enhance psychological functioning to dissociative and anxiety responses with negative acute and long-term sequelae. Ego dissolution may be regarded as a transdiagnostic variable or mechanism (see Kelly et al., 2021), which could be evaluated in diverse psychopathologies (e.g., dissociative disorders, psychotic and schizophrenia spectrum disorders, bipolar disorder, borderline personality disorder). Total EDS score and subscales may prove useful when integrated into psychotherapies that strive to engender loving-kindness and unity with others. We suggest that researchers explore the spectrum of ego dissolution experiences (e.g., experiences of dissolution of body boundaries) and also evaluate high and low scorers on the EDS subscales in terms of psychopathology and responses to psychotherapy, psychedelic drugs, meditation, and other means of inducing ego dissolution.

Studies that explore links among adaptive and maladaptive daydreaming (i.e., excessive, problematic, interferes with functioning, a risk factor for psychopathology; see Somer et al., 2017; Schimmenti et al., 2019 for potential diagnostic criteria for maladaptive daydreaming), dissociation, and ego dissolution would be important to undertake. Just as adaptive vs. maladaptive daydreaming is a meaningful distinction, it is likewise worthwhile to delineate when and under what circumstances ego dissolution is adaptive vs. maladaptive.

## 5 Conclusion

The EDS is a brief, reliable, and cross-validated instrument. Ego dissolution is a broad construct that can be experienced in different ways, by different people, and in different social and cultural contexts. Even within a single psychedelic session, individuals may experience a plethora of experiences of ego dissolution, some positive and some negative, which shift in response to emerging internal and external stimuli. Interviews (see Lindström et al., 2022) that assess the richness, diversity, and dynamic nature of self-loss are necessary to supplement global and dispositional measures to better understand the complex and highly individual phenomenology of ego dissolution experiences on an acute and chronic basis in healthy and clinical populations. To this end, we hope that the EDS will find a place in the armamentarium of clinicians and researchers alike invested in exploring the fascinating and largely uncharted domain of experiences of ego dissolution.

## Data availability statement

The raw data supporting the conclusions of this article will be made available by the authors, without undue reservation.

## Ethics statement

The studies involving humans were approved by Binghamton University (SUNY) Institutional Review Board. The studies were conducted in accordance with the local legislation and institutional requirements. The participants provided their written informed consent to participate in this study.

## Author contributions

SL: Conceptualization, Investigation, Methodology, Project administration, Resources, Supervision, Writing – original draft, Writing – review & editing. CM: Conceptualization, Project administration, Visualization, Writing – original draft, Writing – review & editing. FS: Conceptualization, Data curation, Formal analysis, Investigation, Methodology, Visualization, Writing – original draft, Writing – review & editing. RM: Conceptualization, Data curation, Formal analysis, Supervision, Visualization, Writing – original draft, Writing – review & editing.
