# Supplementary material for: Cross-validation of the ego dissolution scale: implications for studying psychedelics
Source: Front Neurosci. 2023 Dec 5;17:1267611. doi: 10.3389/fnins.2023.1267611 (PMC10729006; doi:10.3389/fnins.2023.1267611)
Supplement: Supplementary file 1 [file Data_Sheet_1.pdf]

1 Supplementary Figures and Tables

Supplementary Figure 1. Ego Dissolution Scale (EDS).

This questionnaire consists of questions about experiences that you may have had in your daily life. We are interested in how often you have these experiences.

To answer the questions, please determine to what degree the experience described in the question applies to you and mark the scale to show what percentage of the time you have the experience.

1. My “self” or ego dissolves into nothingness.

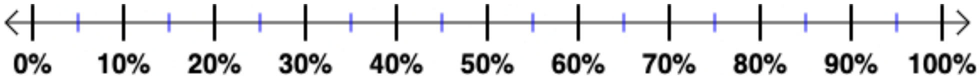

2. My “self” disappears and no “me” or “I” is present any longer.

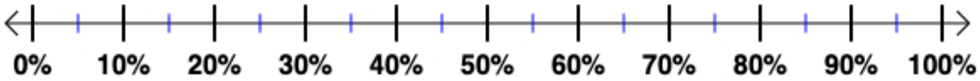

3. I feel I do not exist.

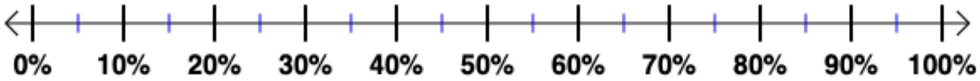

4. I experience being out of my body.

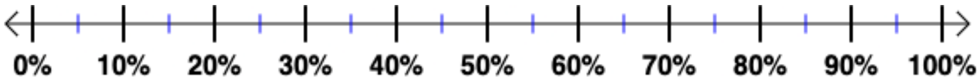

5. I experience a disintegration of my “self” or ego.

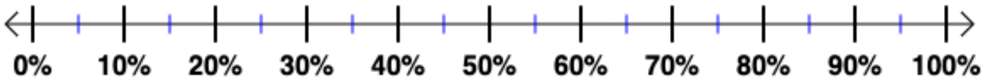

6. My sense of self moved from one part of my body to another (i.e., from behind my eyes to my heart).

0% 10% 20% 30% 40% 50% 60% 70% 80% 90% 100%

7. I feel at one with the universe.

0% 10% 20% 30% 40% 50% 60% 70% 80% 90% 100%

8. I feel one with everything around me.

0% 10% 20% 30% 40% 50% 60% 70% 80% 90% 100%

9. I feel a sense of union with others.

0% 10% 20% 30% 40% 50% 60% 70% 80% 90% 100%

10. I feel I merge with others/the world.

0% 10% 20% 30% 40% 50% 60% 70% 80% 90% 100%

Supplementary Table 1. Correlations among variables

	1	2	3	4	5	6	7	8	9	10	11	12	13
1. Ego dissolution (EDS)	-	-	-	-	-	-	-	-	-	-	-	-	-
2. Ego-Loss (EDS)	.80*	-	-	-	-	-	-	-	-	-	-	-	-
3. Unity (EDS)	.79*	.26*	-	-	-	-	-	-	-	-	-	-	-
4. Trait dissociation (DES-II)	.53*	.63*	.21*	-	-	-	-	-	-	-	-	-	-
5. Trait depersonalization/derealization (DES-II)	.53*	.67*	.16	.84*	-	-	-	-	-	-	-	-	-
6. Pathological dissociation (DES-T)	.54*	.67*	.19*	.91*	.92*	-	-	-	-	-	-	-	-
7. State dissociation (CADSS)	.36*	.50*	.07	.52*	.52*	.51*	-	-	-	-	-	-	-
8. Neuroticism (Mini-IPIP)	.07	.26*	-.16	.30*	.26*	.25*	.25*	-	-	-	-	-	-
9. Extraversion (Mini-IPIP)	.15	-.01	.25*	.01	-.02	-.03	.01	-.03	-	-	-	-	-
10. Intellect/Imagination (Mini-IPIP)	.02	-.01	.04	.01	-.03	-.04	.02	.05	.07	-	-	-	-
11. Agreeableness (Mini-IPIP)	.05	-.09	.17	-.07	-.10	-.11	-.05	.05	.26*	.13	-	-	-
12. Conscientiousness (Mini-IPIP)	-.01	-.14	.12	-.13	-.14	-.15	-.15	-.15	.03	-.01	.12	-	-
13. Positive affect (PANAS)	.26*	.11	.31*	.18	.11	.17	.09	-.15	.19*	.00	-.01	.17	-
14. Negative affect (PANAS)	.27*	.44*	-.02	.43*	.41*	.43*	.32*	.43*	-.01	-.11	-.03	-.17	.24*
15. Trait mindfulness (FFMQ)	-.01	-.26*	.25*	-.30*	-.26*	-.27*	-.24*	-.45*	.18*	.12	.15	.30*	.25*
16. Observing (FFMQ)	.28*	.21*	.24*	.29*	.19*	.21*	.22*	.19*	.09	.17	.16	-.03	.16
17. Describing (FFMQ)	-.02	-.23*	.20*	-.24*	-.23*	-.23*	-.20*	-.20*	.24*	.21*	.22*	.18*	.21*
18. Acting with awareness (FFMQ)	-.20*	-.34*	.02	-.41*	-.33*	-.34*	-.34*	-.38*	.06	-.07	.03	.35*	.11
19. Nonjudging (FFMQ)	-.13	-.27*	.07	-.37*	-.25*	-.30*	-.25*	-.47*	.08	-.02	-.04	.16	.03
20. Nonreactivity (FFMQ)	.11	.02	.16	.06	.00	.05	.03	-.27*	.01	.06	.04	.10	.18*
21. Hallucination proneness (LSHS-R)	.34*	.45*	.09	.54*	.45*	.48*	.37*	.33*	-.01	.08	.03	-.11	.11
22. Transliminality (RTS)	.41*	.40*	.25*	.47*	.36*	.39*	.41*	.21*	.10	.26*	.10	-.07	.13
23. Social desirability (M-C)	.02	-.12	.15	-.15	-.08	-.12	-.18*	-.31*	.00	.00	.13	.23*	.13
24. Neuroticism (NEO-FFI)	.04	.26*	-.21*	.29*	.23*	.24*	.24*	.69*	-.17	.04	.08	-.25*	-.26*
25. Pre-sleep arousal (PSAS-13)	.17	.33*	-.07	.35*	.28*	.27*	.29*	.44*	.02	.06	.02	-.12	-.01
26. Sleep apnea (SLEEP-50)	.25*	.42*	-.04	.41*	.38*	.38*	.32*	.28*	-.05	-.03	-.09	-.18*	.04
27. Insomnia (SLEEP-50)	.17	.30*	-.04	.29*	.23*	.20*	.26*	.40*	-.03	.04	-.01	-.13	.01
28. Narcolepsy (SLEEP-50)	.29*	.47*	-.01	.45*	.46*	.45*	.33*	.32*	-.05	.01	-.11	-.12	.10
29. RLS/PLMD (SLEEP-50)	.26*	.37*	.04	.42*	.41*	.40*	.28*	.23*	-.05	-.08	-.12	-.11	.12

30. Circadian rhythm dysfunction (SLEEP-50)	.13	.23*	-.03	.25*	.21*	.21*	.20*	.20*	-.08	.03	-.03	-.13	.00
31. Sleep walking (SLEEP-50)	.33*	.41*	.11	.38*	.40*	.43*	.19*	.10	.00	-.09	-.13	-.07	.25*
32. Nightmares (SLEEP-50)	.19*	.26*	.03	.29*	.22*	.23*	.21*	.27*	-.03	.11	.03	-.07	.07
33. Factors affecting sleep quality (SLEEP-50)	.23*	.40*	-.04	.36*	.39*	.36*	.35*	.40*	-.02	.04	-.01	-.23*	-.07
34. Functional impairment due to poor sleep (SLEEP-50)	.10	.27*	-.11	.31*	.22*	.21*	.33*	.46*	-.08	.06	.01	-.25*	-.12

	14	15	16	17	18	19	20	21	22	23	24	25	26
1. Ego dissolution (EDS)	-	-	-	-	-	-	-	-	-	-	-	-	-
2. Ego-Loss (EDS)	-	-	-	-	-	-	-	-	-	-	-	-	-
3. Unity (EDS)	-	-	-	-	-	-	-	-	-	-	-	-	-
4. Trait dissociation (DES-II)	-	-	-	-	-	-	-	-	-	-	-	-	-
5. Trait depersonalization/derealization (DES-II)	-	-	-	-	-	-	-	-	-	-	-	-	-
6. Pathological dissociation (DES-T)	-	-	-	-	-	-	-	-	-	-	-	-	-
7. State dissociation (CADSS)	-	-	-	-	-	-	-	-	-	-	-	-	-
8. Neuroticism (Mini-IPIP)	-	-	-	-	-	-	-	-	-	-	-	-	-
9. Extraversion (Mini-IPIP)	-	-	-	-	-	-	-	-	-	-	-	-	-
10. Intellect/Imagination (Mini-IPIP)	-	-	-	-	-	-	-	-	-	-	-	-	-
11. Agreeableness (Mini-IPIP)	-	-	-	-	-	-	-	-	-	-	-	-	-
12. Conscientiousness (Mini-IPIP)	-	-	-	-	-	-	-	-	-	-	-	-	-
13. Positive affect (PANAS)	-	-	-	-	-	-	-	-	-	-	-	-	-
14. Negative affect (PANAS)	-	-	-	-	-	-	-	-	-	-	-	-	-
15. Trait mindfulness (FFMQ)	-.33*	-	-	-	-	-	-	-	-	-	-	-	-
16. Observing (FFMQ)	.17	.21	-	-	-	-	-	-	-	-	-	-	-
17. Describing (FFMQ)	-.20	.74*	.17	-	-	-	-	-	-	-	-	-	-
18. Acting with awareness (FFMQ)	-.38*	.60*	-.37*	.33*	-	-	-	-	-	-	-	-	-
19. Nonjudging (FFMQ)	-.33*	.62*	-.37*	.25*	.54*	-	-	-	-	-	-	-	-
20. Nonreactivity (FFMQ)	-.05	.44*	.34*	.23*	-.12	-.04	-	-	-	-	-	-	-
21. Hallucination proneness (LSHS-R)	.35*	-.28*	.35*	-.18	-.43*	-.45*	.12	-	-	-	-	-	-
22. Transliminality (RTS)	.20*	-.10	.39*	-.06	-.35*	-.27*	.15	.49*	-	-	-	-	-
23. Social desirability (M-C)	-.17	.33*	-.12	.19	.33*	.34*	.05	-.18	-.11	-	-	-	-
24. Neuroticism (NEO-FFI)	.45*	-.54*	.25*	-.28*	-.54*	-.57*	-.17	.35*	.24*	-.32*	-	-	-

25. Pre-sleep arousal (PSAS-13)	.39*	-.31*	.33*	-.13	-.45*	-.43*	-.03	.38*	.34*	-.24*	.47*	-	-
26. Sleep apnea (SLEEP-50)	.35*	-.25*	.15	-.18	-.29*	-.29*	.03	.31*	.25*	-.17	.28*	.45*	-
27. Insomnia (SLEEP-50)	.34*	-.25*	.27*	-.11	-.38*	-.36*	.02	.32*	.26*	-.24*	.44*	.62*	.48*
28. Narcolepsy (SLEEP-50)	.43*	-.20	.24*	-.14	-.28*	-.27*	.03	.40*	.30*	-.12	.31*	.26*	.52*
29. RLS/PLMD (SLEEP-50)	.32*	-.25*	.14	-.20	-.27*	-.26*	.02	.40*	.23*	-.12	.31*	.36*	.52*
30. Circadian rhythm dysfunction (SLEEP-50)	.26*	-.17	.18	-.08	-.28*	-.24*	.07	.28*	.14	-.16	.25*	.33*	.38*
31. Sleep walking (SLEEP-50)	.36*	-.08	.07	-.08	-.11	-.13	.10	.21*	.16	.01	.30*	.32*	.43*
32. Nightmares (SLEEP-50)	.27*	-.12	.27*	-.03	-.25*	-.31*	.12	.32*	.30*	-.12	.06	.19	.45*
33. Factors affecting sleep quality (SLEEP-50)	.38*	-.27*	.23*	-.16	-.36*	-.33*	-.02	.34*	.27*	-.23*	.28*	.39*	.49*
34. Functional impairment due to poor sleep (SLEEP-50)	.36*	-.33*	.29*	-.18	-.51*	-.39*	.03	.29*	.26*	-.31*	.42*	.42*	.41*

	27	28	29	30	31	32	33	34
1. Ego dissolution (EDS)	-	-	-	-	-	-	-	-
2. Ego-Loss (EDS)	-	-	-	-	-	-	-	-
3. Unity (EDS)	-	-	-	-	-	-	-	-
4. Trait dissociation (DES-II)	-	-	-	-	-	-	-	-
5. Trait depersonalization/derealization (DES-II)	-	-	-	-	-	-	-	-
6. Pathological dissociation (DES-T)	-	-	-	-	-	-	-	-
7. State dissociation (CADSS)	-	-	-	-	-	-	-	-
8. Neuroticism (Mini-IPIP)	-	-	-	-	-	-	-	-
9. Extraversion (Mini-IPIP)	-	-	-	-	-	-	-	-
10. Intellect/Imagination (Mini-IPIP)	-	-	-	-	-	-	-	-
11. Agreeableness (Mini-IPIP)	-	-	-	-	-	-	-	-
12. Conscientiousness (Mini-IPIP)	-	-	-	-	-	-	-	-
13. Positive affect (PANAS)	-	-	-	-	-	-	-	-
14. Negative affect (PANAS)	-	-	-	-	-	-	-	-
15. Trait mindfulness (FFMQ)	-	-	-	-	-	-	-	-
16. Observing (FFMQ)	-	-	-	-	-	-	-	-
17. Describing (FFMQ)	-	-	-	-	-	-	-	-
18. Acting with awareness (FFMQ)	-	-	-	-	-	-	-	-
19. Nonjudging (FFMQ)	-	-	-	-	-	-	-	-

20. Nonreactivity (FFMQ)	-	-	-	-	-	-	-	-
21. Hallucination proneness (LSHS-R)	-	-	-	-	-	-	-	-
22. Transliminality (RTS)	-	-	-	-	-	-	-	-
23. Social desirability (M-C)	-	-	-	-	-	-	-	-
24. Neuroticism (NEO-FFI)	-	-	-	-	-	-	-	-
25. Pre-sleep arousal (PSAS-13)	-	-	-	-	-	-	-	-
26. Sleep apnea (SLEEP-50)	-	-	-	-	-	-	-	-
27. Insomnia (SLEEP-50)	-	-	-	-	-	-	-	-
28. Narcolepsy (SLEEP-50)	.40*	-	-	-	-	-	-	-
29. RLS/PLMD (SLEEP-50)	.36*	.55*	-	-	-	-	-	-
30. Circadian rhythm dysfunction (SLEEP-50)	.38*	.42*	.34*	-	-	-	-	-
31. Sleep walking (SLEEP-50)	.18	.48*	.53*	.24*	-	-	-	-
32. Nightmares (SLEEP-50)	.42*	.42*	.37*	.30*	.29*	-	-	-
33. Factors affecting sleep quality (SLEEP-50)	.46*	.46*	.36*	.39*	.36*	.34*	-	-
34. Functional impairment due to poor sleep (SLEEP-50)	61*	.38*	26*	.53*	.08	.37*	.46*	-

Note. (n = 527). CADSS = Clinician-Administered Dissociative State Scale; DES-II = Dissociative Experiences Scale II; EDS = Ego Dissolution Scale; FFMQ = Five Facet Mindfulness Scale; LSHS-R = Launay-Slade Hallucination Scale-Revised; M-C = Marlowe-Crowne Social Desirability Scale Form C; NEO-FFI = NEO Five-Factor Inventory; PANAS = Positive and Negative Affect Schedule; PLMD = Periodic Leg Movement Disorder; PSAS-13 = Pre-sleep Arousal Scale; RLS = Restless Leg Syndrome; RTS = Revised Transliminality Scale

* = $p < .001$ (with Bonferroni correction; $p < .00000082$)

Supplementary Table 2. Correlations between Ego-Loss (EDS), Unity (EDS), and other variables of interest and differences between correlation coefficients

Variable	Ego-Loss (EDS)	Unity (EDS)	Steiger's *z*
Trait dissociation (DES-II)	.63*	.21	9.34*
Trait depersonalization/derealization (DES-II)	.67*	.16	11.46*
Pathological dissociation (DES-T)	.67*	.19	10.88*
State dissociation (CADSS)	.50*	.07	8.76*
Neuroticism (Mini-IPIP)	.26*	-.16	8.04*
Extraversion (Mini-IPIP)	-.01	.25*	-4.97*
Intellect/Imagination (Mini-IPIP)	-.01	.04	-0.94
Agreeableness (Mini-IPIP)	-.09	.17	-4.93*
Conscientiousness (Mini-IPIP)	-.14	.12	-4.92*
Positive affect (PANAS)	.11	.31*	-4.09*
Negative affect (PANAS)	.44*	-.02	9.12*
Trait mindfulness (FFMQ)	-.26*	.25*	-9.81*
Observing (FFMQ)	.21	.24*	-0.59
Describing (FFMQ)	-.23*	.20	-8.22*
Acting with awareness (FFMQ)	-.34*	.02	-6.98*
Nonjudging (FFMQ)	-.27*	.07	-6.51*
Nonreactivity (FFMQ)	.02	.16	-2.46
Hallucination proneness (LSHS-R)	.45*	.09	7.24*
Transliminality (RTS)	.40*	.25*	3.06
Social desirability (M-C)	-.12	.15	-5.11*
Neuroticism (NEO-FFI)	.26*	-.21	9.02*
Pre-sleep arousal (PSAS-13)	.33*	-.07	7.73*
Sleep apnea (SLEEP-50)	.42*	-.04	9.06*
Insomnia (SLEEP-50)	.30*	-.04	6.54*
Narcolepsy (SLEEP-50)	.47*	-.01	9.60*
RLS/PLMD (SLEEP-50)	.37*	.04	6.46*
Circadian rhythm dysfunction (SLEEP-50)	.23*	-.03	4.96*
Sleep walking (SLEEP-50)	.41*	.11	5.98*
Nightmares (SLEEP-50)	.26*	.03	4.41*
Factors affecting sleep quality (SLEEP-50)	.40*	-.04	8.63*
Functional impairment due to poor sleep (SLEEP-50)	.27*	-.11	7.27*

Note. (n = 527). CADSS = Clinician-Administered Dissociative State Scale; DES-II = Dissociative Experiences Scale II; DES-T = Dissociative Experiences Scale – Taxon; EDS = Ego Dissolution Scale; FFMQ = Five Facet Mindfulness Scale; LSHS-R = Launay-Slade Hallucination Scale-Revised; M-C = Marlowe-Crowne Social Desirability Scale Form C; NEO-FFI = NEO Five-Factor Inventory; PANAS = Positive and Negative Affect Schedule; PLMD = Periodic Leg Movement Disorder; PSAS-13 = Pre-sleep Arousal Scale; RLS = Restless Leg Syndrome; RTS = Revised Transliminality Scale

* = $p < .001$ (with Bonferroni correction; $p < .00000082$)

Supplementary Table 3. Correlations between trait depersonalization/derealization (DES-II) and Ego-Loss (EDS) and trait depersonalization/derealization with Unity (EDS), and other variables of interest and differences between correlation coefficients

Variable	Trait depersonalization/derealization (DES-II)	Ego-Loss (EDS)	Steiger's *z*	Unity (EDS)	Steiger's *z*
State dissociation (CADSS)	.52	.50*	-0.68	.07	-8.69*
Neuroticism (Mini-IPIP)	.26*	.26*	0.00	-.16	-7.54*
Extraversion (Mini-IPIP)	-.02	-.01	0.28	.25*	4.84*
Intellect/Imagination (Mini-IPIP)	-.03	-.01	0.56	.05	1.41
Agreeableness (Mini-IPIP)	-.10	-.09	0.28	.17	4.80*
Conscientiousness (Mini-IPIP)	-.14	-.14	0.00	.12	4.62*
Positive affect (PANAS)	.11	.11	0.00	.31*	3.66*
Negative affect (PANAS)	.41*	.44*	0.95	-.02	-7.95*
Trait mindfulness (FFMQ)	-.26*	-.26*	0.00	.25*	9.21*
Observing (FFMQ)	.19	.21	0.58	.24*	0.91
Describing (FFMQ)	-.23*	-.23*	0.00	.20	7.72*
Acting with awareness (FFMQ)	-.33*	-.34*	-0.30	.02	6.36*
Nonjudging (FFMQ)	-.25*	-.27*	-0.59	.07	5.73*
Nonreactivity (FFMQ)	.00	.02	0.56	.16	2.84
Hallucination proneness (LSHS-R)	.45*	.45*	0.00	.09	-6.81*
Transliminality (RTS)	.36*	.40*	1.24	.25*	-2.09
Social desirability (M-C)	-.08	-.12	-1.13	.15	4.08*
Neuroticism (NEO-FFI)	.23*	.26*	0.88	-.21	-7.90*
Pre-sleep arousal (PSAS-13)	.28*	.33*	1.49	-.07	-6.30*
Sleep apnea (SLEEP-50)	.38*	.42*	1.25	-.04	-7.70*
Insomnia (SLEEP-50)	.23*	.30*	2.06	-.04	-4.83*
Narcolepsy (SLEEP-50)	.46*	.47*	0.33	-.01	-8.81*
RLS/PLMD (SLEEP-50)	.41*	.37*	-1.24	.04	-6.88*
Circadian rhythm dysfunction (SLEEP-50)	.21	.23*	0.58	-.03	-4.28*
Sleep walking (SLEEP-50)	.40*	.41*	0.31	.11	-5.42*
Nightmares (SLEEP-50)	.22*	.26*	1.17	.03	-3.40
Factors affecting sleep quality (SLEEP-50)	.39*	.40*	0.31	-.04	-7.90*

Functional impairment due to poor sleep (SLEEP-50)	.22*	.27*	1.46	-.11	-5.90*

Note. (n = 527). CADSS = Clinician-Administered Dissociative State Scale; DES-II = Dissociative Experiences Scale II; EDS = Ego Dissolution Scale; FFMQ = Five Facet Mindfulness Scale; LSHS-R = Launay-Slade Hallucination Scale-Revised; M-C = Marlowe-Crowne Social Desirability Scale Form C; NEO-FFI = NEO Five-Factor Inventory; PANAS = Positive and Negative Affect Schedule; PLMD = Periodic Leg Movement Disorder; PSAS-13 = Pre-sleep Arousal Scale; RLS = Restless Leg Syndrome; RTS = Revised Transliminality Scale

* = $p < .001$ (with Bonferroni correction; $p < .00000082$)

Supplementary Table 4. Multiple linear regression predicting EDS total score

**Model**	***B***	***SE(B)***	***β***	***t***	***p***
Outcome: EDS total score					
Constant	1.342	3.901		.344	.731
Pathological dissociation (DES-T)	.267	.087	.257	3.071	.002
Transliminality (RTS)	.682	.136	.203	5.017	.000
Extraversion (Mini-IPIP)	.497	.140	.128	3.558	.000
Conscientiousness (Mini-IPIP)	.474	.172	.100	2.758	.006
Observing (FFMQ)	.318	.089	.136	3.549	.000
Neuroticism (NEO-FFI)	-.209	.061	-.135	-3.446	.001
Derealization/depersonalization (DES-II)	.228	.077	.243	2.964	.003
Model R^2 = .412					

Note. (n = 492). DES-II = Dissociative Experiences Scale II; DES-T = Dissociative Experiences Scale – Taxon; FFMQ = Five Facet Mindfulness Questionnaire; Mini-IPIP = Mini International Personality Item Pool; NEO-FFI = NEO Five-Factor Inventory; and RTS = Revised Transliminality Scale.

Supplementary Table 5. Multiple linear regression predicting Ego-Loss

**Model**	***B***	***SE(B)***	***β***	***t***	***p***
Outcome: Ego-Loss					
Constant	1.338	2.185		.612	.541
Derealization/depersonalization (DES-II)	.420	.062	.438	6.746	.000
State dissociation (CADSS)	.269	.059	.159	4.552	.000
Negative affect (PANAS)	.189	.056	.107	3.350	.001
Circadian rhythm dysfunction (SLEEP-50)	.570	.190	.091	2.994	.003
Describing (FFMQ)	-.158	.064	-.074	-2.472	.014
Pathological dissociation (DES-T)	.167	.068	.159	2.466	.014
Model R^2 = .611					

Note. (*n* = 480). CADSS = Clinician-Administered Dissociative State Scale; DES-II = Dissociative Experiences Scale II; DES-T = Dissociative Experiences Scale – Taxon; FFMQ = Five Facet Mindfulness Questionnaire; PANAS = Positive and Negative Affect Schedule; and SLEEP-50 = SLEEP-50 Questionnaire.

Supplementary Table 6. Multiple linear regression predicting Unity

**Model**	***B***	***SE(B)***	***β***	***t***	***p***
Outcome: Unity					
Constant	-11.938	6.437		-1.855	.064
Positive affect (PANAS)	.497	.109	.188	4.576	.000
Transliminality (RTS)	.889	.242	.161	3.675	.000
Extraversion (Mini-IPIP)	1.394	.256	.213	5.434	.000
Pathological dissociation (DES-T)	.366	.071	.230	5.184	.000
Neuroticism (Mini-IPIP)	-1.014	.343	-.134	-2.975	.003
Observing (FFMQ)	.796	.163	.201	4.873	.000
Social desirability (M-C)	.941	.335	.112	2.810	.005
Pre-sleep arousal (PSAS-13)	-.252	.098	-.116	-2.572	.010
Negative affect (PANAS)	-.300	.137	-.103	-2.198	.028
Agreeableness (Mini-IPIP)	.693	.342	.081	2.024	.043
Model R^2 = .346					

Note. (*n* = 497). DES-T = Dissociative Experiences Scale – Taxon; FFMQ = Five Facet Mindfulness Questionnaire; M-C = Marlowe-Crowne Social Desirability Scale Form C; Mini-IPIP = Mini International Personality Item Pool; PANAS = Positive and Negative Affect Schedule; PSAS-13 = Pre-sleep Arousal Scale; and RTS = Revised Transliminality Scale.
